# Evaluation of Large Language Model Performance and Reliability for Citations and References in Scholarly Writing: Cross-Disciplinary Study

**DOI:** 10.2196/52935

**Published:** 2024-04-05

**Authors:** Joseph Mugaanyi, Liuying Cai, Sumei Cheng, Caide Lu, Jing Huang

**Affiliations:** 1 Department of Hepato-Pancreato-Biliary Surgery, Ningbo Medical Center Lihuili Hospital Health Science Center Ningbo University Ningbo China; 2 Institute of Philosophy Shanghai Academy of Social Sciences Shanghai China

**Keywords:** large language models, accuracy, academic writing, AI, cross-disciplinary evaluation, scholarly writing, ChatGPT, GPT-3.5, writing tool, scholarly, academic discourse, LLMs, machine learning algorithms, NLP, natural language processing, citations, references, natural science, humanities, chatbot, artificial intelligence

## Abstract

**Background:**

Large language models (LLMs) have gained prominence since the release of ChatGPT in late 2022.

**Objective:**

The aim of this study was to assess the accuracy of citations and references generated by ChatGPT (GPT-3.5) in two distinct academic domains: the natural sciences and humanities.

**Methods:**

Two researchers independently prompted ChatGPT to write an introduction section for a manuscript and include citations; they then evaluated the accuracy of the citations and Digital Object Identifiers (DOIs). Results were compared between the two disciplines.

**Results:**

Ten topics were included, including 5 in the natural sciences and 5 in the humanities. A total of 102 citations were generated, with 55 in the natural sciences and 47 in the humanities. Among these, 40 citations (72.7%) in the natural sciences and 36 citations (76.6%) in the humanities were confirmed to exist (*P*=.42). There were significant disparities found in DOI presence in the natural sciences (39/55, 70.9%) and the humanities (18/47, 38.3%), along with significant differences in accuracy between the two disciplines (18/55, 32.7% vs 4/47, 8.5%). DOI hallucination was more prevalent in the humanities (42/55, 89.4%). The Levenshtein distance was significantly higher in the humanities than in the natural sciences, reflecting the lower DOI accuracy.

**Conclusions:**

ChatGPT’s performance in generating citations and references varies across disciplines. Differences in DOI standards and disciplinary nuances contribute to performance variations. Researchers should consider the strengths and limitations of artificial intelligence writing tools with respect to citation accuracy. The use of domain-specific models may enhance accuracy.

## Introduction

### Background

In the ever-evolving landscape of scholarly research and academic discourse, the role of technology in aiding and enhancing the research process has grown exponentially. One of the most notable advancements in this regard is the emergence of large language models (LLMs) such as GPT-3.5, which have demonstrated impressive capabilities in generating written content across various domains, including academic writing. These LLMs, powered by vast corpora of text data and sophisticated machine-learning algorithms, have offered researchers and writers a new tool for assistance in crafting scholarly documents [1-3]. LLMs were initially designed and developed to primarily assist in natural language writing. However, since the release of ChatGPT in late 2022, the tool has been adopted in a wide range of scenarios, including customer care, expert systems, as well as literature searches and academic writing. Researchers have already used LLMs to write their academic papers, as demonstrated by Kishony and Ifargan [4]. While the potential of these tools is evident, it is essential to critically assess their performance, especially in the intricate domains of citations and references, which are the foundation of academic discourse and credibility.

Citations and references serve as the backbone of scholarly communication, providing the necessary context, evidence, and credit to prior works, thus fostering intellectual dialogue and ensuring the integrity of the research process. Accuracy in generating citations and the inclusion of Digital Object Identifiers (DOIs) [5] are paramount, as they directly influence the traceability and accessibility of cited works. Despite the promise of LLMs, concerns have emerged regarding the reliability and precision of their generated citations and references, raising questions about their suitability as academic writing assistants. Studies on the viability of LLMs as writing assistants in scholarly writing [6-8] underscore the significance of this body of research within the broader academic landscape. Although prior works are quite informative [9-12], there is a lack of an interdisciplinary perspective on citations and references generated by LLMs, which is vital for understanding how LLMs perform across different disciplines.

An increasing number of academics and researchers, especially in countries where English is not a first language (eg, China), are relying on ChatGPT to translate their work into English, research the existing published literature, and even generate citations and references to published literature. Therefore, the aim of this study was to evaluate LLM performance in generating citations and references across two distinct domains, the natural sciences and humanities, by assessing both the presence and accuracy of citations, the existence and accuracy of DOIs, and the potential for hallucination. We aim to provide valuable insights into the strengths and limitations of LLMs in supporting academic writing in diverse research contexts.

The outcomes of this study will contribute to a nuanced understanding of the capabilities and limitations of LLMs as academic writing assistants. Moreover, our findings may inform best practices for researchers and writers who employ these tools in their work, fostering transparency and accuracy in scholarly communication.

### LLM Concepts

An LLM is a catch-all term for a machine-learning model designed and trained to understand and generate natural language. LLMs are considered “large” language models due to the sheer number of parameters in the model. A parameter in machine learning is a numerical variable or weight that is optimized through training to map a relationship between the input and the output. LLMs have millions to billions of parameters.

**Figure 1:**
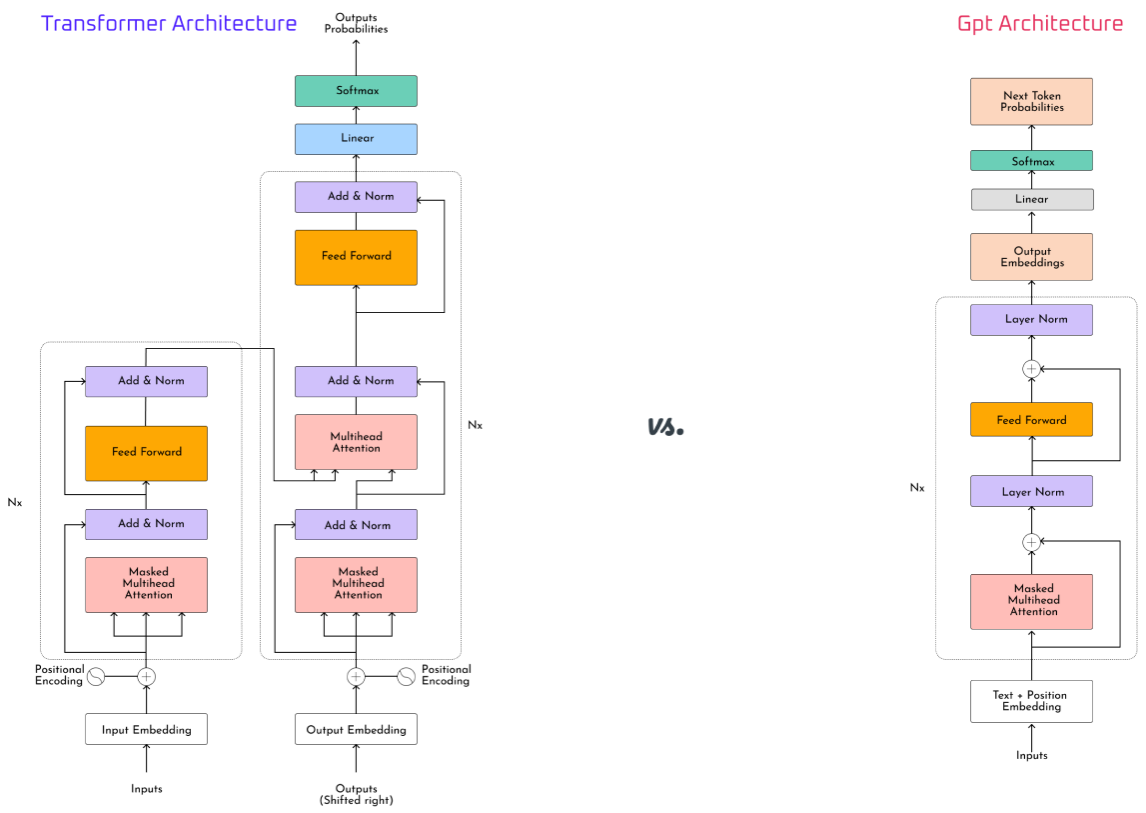
Transformer model architecture (left) and GPT architecture (right).

Current LLMs are mostly based on the transformer architecture (Figure 1). However, before transformers were introduced in 2017 [13], recurrent neural nets (RNNs) were mostly used for natural language processing. One key limitation of RNNs was the length of text they could handle. In 2015, Bahdanau et al [14] proposed accounting for attention to improve RNN performance with long text. Drawing inspiration for the RNN’s encoder-decoder design, the transformer consists of an encoder and a decoder; however, unlike the RNN, the transformer does not perform sequential data processing and each layer can address all other layers. This allows the transformer model to handle different parts of the input as it processes each part at different stages. This is the mechanism that allows for self-attention in the transformer model.

The way attention works in a transformer model is by computing attention weights for each token, and then the relevance of the token is determined based on the weights. This allows the model to track and assign hierarchical values to each token. Fundamentally, this is similar to how humans process language by extracting the key details out of a chunk of text. This architecture is the linchpin for the majority of LLMs, including the GPT model [15] that is the basis of OpenAI’s ChatGPT or the bidirectional encoder representations from transformers (BERT) algorithm [16]. These are broadly categorized into encoder-style and decoder-style transformers, with the former mostly applying to predictive tasks and the latter applying to generative tasks.

Irrespective of the architecture, as an encoder-style or decoder-style transformer, the model is trained on a vast volume of data. The objective is to train a model capable of applying the knowledge gained from the training data to unseen data or situations. This is referred to as generalization. If the model is capable of precise recall of data it has previously been exposed to, this would be memorization and overfitting is said to have occurred. However, this does not mean that memorization is in itself a negative feature. Indeed, there are situations where memorization is preferable to generation such as in the task of information cataloging.

### LLMs in Academia

LLMs can handle tasks such as text classification, translation, summarization, and text generation. Since the advent of the internet, and with it the publication of scientific information online, the amount of global academic output exploded, with more than 5 million articles published in 2022 (Table 1). Given the pressure in academia to keep up with developments in one’s field, it is increasingly becoming more difficult to track, prioritize, and keep up with scientific information. It is against this backdrop that LLMs offer an opportunity. Perhaps the most obvious use case is in literature reviews and summarization, reference lookup, and data generation.

**Table 1:** Number of academic papers published per year, 2018-2022.

Year	Number of articles published (millions)
2018	4.18
2019	4.43
2020	4.68
2021	5.03
2022	5.14

However, there are still several questions that need to be answered. First, machine-learning models are inherently probabilistic, meaning that they are not deterministic. Therefore, for the same user input, the model may give different results due to the variability baked into the model. While this can be a valuable trait for creative endeavors, in academic and scientific works, there is a need for reproducibility and reliability, and it remains unclear how well this can be achieved. Second, LLMs are constrained to the information they are trained on. This can be affected by selection bias, the quality of data used, artifacts resulting from data cleaning, and other factors. In essence, we rely on trusting the trainer to provide accurate and unbiased training data to the models.

There is potential for LLMs to be useful tools for delivering academic and scientific information to various audiences, including—but not limited to—students and other academics. However, for this use case, a degree of memorization of the underlying content is necessary. Where information is unviable, it would be better to state so rather than to interpolate. In the current iteration of LLMs, since the training is geared toward generalization and the models are probabilistic, they tend to interpolate and fill in the missing information with synthetic text. There is still a need to explore this process deeper to find solutions.

## Methods

### Data Collection and Validation

Topics were selected and categorized as either natural sciences or humanities. Topics were included if they were: (1) clinical or biomedical–related research in the natural sciences category and philosophy/psychology-related research in the humanities category, and (2) published in English. Topics were excluded if they were: (1) not in English, (2) related to a highly specialized or niche field, and (3) sensitive or controversial in nature. Two researchers independently prompted ChatGPT (GPT-3.5) to write sections of a manuscript while adhering to the American Psychological Association style [17] for citations and including the DOI of each reference. Citations and references generated by ChatGPT were collected for subsequent analysis. The researchers then independently validated the references by conducting searches on Google Scholar, PubMed, and Google Search for each cited reference. The primary objective was to confirm the existence and accuracy of the cited literature. DOI existence and validation were confirmed using the DOI Foundation website [18]. DOIs that did not exist or were matched to a different source were considered hallucinations [19]. Data collected by both researchers were aggregated and compared. Independent validation was performed to ensure agreement between the two researchers regarding the existence, validity, and accuracy of the citations and DOIs. Any disagreements or discrepancies were resolved through discussion and consensus.

In this study, hallucination refers to instances where ChatGPT 3.5 generates DOIs and/or citations that do not correspond to actual, valid DOIs/citations for scholarly references. In these instances, the model may produce DOIs and/or citations that seem authentic but are in fact incorrect or nonexistent. The Levenshtein distance, also known as the edit distance, is a measure of the similarity between two strings by calculating the minimum number of single-character edits (insertions, deletions, or substitutions) required to transform one string into the other. In other words, this metric quantifies the “distance” between two strings in terms of the minimum number of operations needed to make them identical. We used the Levenshtein distance to compare the DOI generated by ChatGPT with the correct DOI. This comparison helps to measure how closely the artificial intelligence (AI)–generated DOI aligns with the expected DOI for a given citation. By calculating the Levenshtein distance, we can quantify the differences between the AI-generated DOI and the correct DOI. Larger Levenshtein distance values suggest greater dissimilarity, indicating potential inaccuracies in the AI-generated DOI.

### Statistical Analysis

Data analysis was conducted using SPSS 26 and Python. The Levenshtein distance [20] between the generated DOI and the actual DOI was calculated using the *thefuzz* package in Python to quantitatively assess the DOI accuracy. Continuous variables are reported as mean (SD) and categorical variables are presented as absolute numbers and percentages. An independent-sample *t* test was used to compare continuous variables, whereas the Fisher exact test was used for comparisons of categorical variables. A *P* value <.05 was considered statistically significant in all tests.

### Ethical Considerations

This study was exempt from ethical review since no animal or human participants were involved.

## Results

### Included Topics and Citations

Ten manuscript topics were selected and included in the study, with 5 in the natural sciences group and 5 in the humanities group. ChatGPT 3.5 was prompted to write an introduction section for each topic between July 10 and August 15, 2023. A total of 102 citations were generated by ChatGPT. Of these, 55 were in the natural sciences group and 47 in the humanities group. The existence, validity, and relevance of citations were examined irrespective of the corresponding DOIs. The results are summarized in Table 2. A list of the included topics and a sample of prompts to ChatGPT are provided in Multimedia Appendix 1.

**Table 2:** Data analysis results.

Variables	Natural sciences (n=55)	Humanities (n=47)	*P* value^a^
Citation exists, n (%)	40 (72.7)	36 (76.6)	.42
Citation accurate, n (%)	37 (67.3)	29 (61.7)	.35
Relevant, n (%)	39 (70.9)	35 (74.5)	.43
DOI^b^ exists, n (%)	39 (70.9)	18 (38.3)	.001
DOI accurate, n (%)	18 (32.7)	4 (8.5)	.003
DOI hallucination, n (%)	34 (61.8)	42 (89.4)	.001
Levenshtein distance, mean (SD)	64.13 (42.26)	42.15 (40.23)	.009

^a^Categorical variables were compared using the Fisher exact test; the continuous variable (Levenshtein distance) was compared using the independent-sample *t* test.

^b^DOI: Digital Object Identifier.

### Citation Existence and Accuracy

Of the 102 generated citations, 76 (74.5%) were found to be real and exist in the published literature, with 72.7% and 76.6% of the citations verified in the natural and humanities group, respectively. There was no significant difference between the two groups (*P*=.42), indicating that the validity of the citations was relatively consistent between the two domains. Similarly, when assessing the accuracy of the citations, no significant difference was observed (Table 2).

### Citation Relevance

The relevance of citations generated by ChatGPT was evaluated by assessing whether they were appropriate and contextually meaningful within the research topics. Our analysis indicated that 70.9% and 74.5% of citations in the natural sciences and humanities categories were deemed relevant, respectively (Table 2). The difference was not statistically significant (*P*=.43), suggesting that ChatGPT demonstrated a similar ability to generate contextually relevant citations in both domains.

### DOI Existence, Accuracy, and Hallucination

Our analysis revealed significant differences between the two domains with respect to DOIs. In the natural sciences, 70.9% of the included DOIs were real, whereas in the humanities, only 38.3% of the DOIs generated were real (*P*=.001; Table 2). Similarly, the level of DOI accuracy was significantly higher for the natural sciences than for the humanities (*P*=.003). Moreover, the occurrence of DOI hallucination, where ChatGPT generates DOIs that do not correspond with the existing literature, was more prevalent in the humanities than in the natural sciences (*P*=.001). The mean Levenshtein distance, which measures the deviation between the generated DOI and the actual DOI, was significantly higher in the natural sciences group than in the humanities (*P*=.009; Table 2).

## Discussion

### Principal Findings

The results of this study shed light on the performance of ChatGPT (GPT-3.5) as an academic writing assistant in generating citations and references in natural sciences and humanities topics. Our findings reveal notable differences in the accuracy and reliability of the citations and references generated by ChatGPT when applied to natural sciences and humanities topics. Hallucination in the context of LLMs such as ChatGPT refers to a phenomenon where the model generates content that is incorrect, fabricated, or not grounded in reality. Hallucination occurs when the model produces information that appears plausible or contextually relevant but lacks accuracy or fidelity to real-world knowledge.

The most striking observation was the significant disparity in the existence and accuracy of the DOIs between the two domains. In natural sciences topics, DOIs were real in 70.9% of the generated citations, representing a significantly higher rate compared to the low rate of 38.3% real DOIs in the humanities topics. The discrepancies in the DOI existence and accuracy in the two domains may be attributed to the differential adoption and availability of DOIs across academic disciplines, where the natural sciences literature has often been more proactive in adopting the DOI system of referencing and linking to scholarly works than the humanities. It is a general practice that journals publishing on the natural sciences frequently mandate DOI inclusion, whereas publishers in the humanities have been slower to adopt such standards [21,22]. Consequently, the performance of the ChatGPT LLM in generating accurate DOIs appears to reflect these disciplinary disparities.

LLMs may generate fictional “facts” presented as true “real-world facts,” which is referred to as hallucination [19,23]. In this study, we considered hallucination to have occurred if the DOI of the generated citation was not real or was real but was linked to a different source. DOI hallucination was more frequent in the humanities (89.4%) than in the natural sciences (61.8%). This finding may be explained by the broader and less structured nature of the humanities literature. There is also a high tendency to provide citations from books and other media that do not use DOIs in the humanities. Therefore, researchers in the humanities should not consider DOIs generated by ChatGPT. Even when ChatGPT generates DOIs for humanities citations, they are more likely to deviate from the correct DOI, potentially leading to the inability to access the cited sources and use the DOIs in citation management tools such as EndNote.

In contrast to the disparities observed in DOI-related metrics, our study found a remarkable consistency in the existence, validity, and relevance of the generated citations in the natural sciences and humanities, with real citations found 72.7% and 76.6% of the time and accurate citations confirmed in 67.3% and 61.7% of cases, respectively. This suggests that the citations generated by ChatGPT can be expected to be reliable approximately 60% of the time.

The divergent performance of ChatGPT between the natural sciences and humanities underscores the importance of considering disciplinary nuances when implementing AI-driven writing assistants in academic contexts. Researchers and writers in both domains should be aware of the strengths and limitations of such tools, particularly in relation to citation practices and DOI accuracy. Future research could delve deeper into the factors influencing DOI accuracy and explore strategies for improving DOI generation by LLMs in the humanities literature. Additionally, the development of domain-specific AI writing models may offer tailored solutions to enhance citation and reference accuracy in various academic disciplines.

In this study, we focused only on the potential use of LLMs in citations and references in scholarly writing; however, the scope to which these models are going to be adopted in academic works is much broader. We believe that these models will be improved over time and that they are here to stay. As such, our argument in this paper is not that LLMs should not be used in scholarly writing, but rather that in their iteration, we ought to be aware of their limitations, primarily concerning the reliability of not only the text they generate but also how they interpret that text.

Although the transformer models that are the foundation of LLMs are very capable of handling a significant amount of information, they still do have context-window limitations. The context window is the textual range or span of the input that the LLM can evaluate to generate a response at any given moment. As an example, GPT-3 has a context window of 2000 tokens, whereas GPT-4’s context window is 32,000 tokens. As such, since the size of the context window impacts model performance (larger is better), GPT-4 outperforms GPT-3 (at the cost of more computation and memory). In scientific knowledge, context is key. Removing a word from the context may greatly affect the information being conveyed. Therefore, we believe that the future of LLMs in academia will rely on fine-tuning the LLMs to capitalize on memorization where necessary, reproducibility and stability of the models, as well as access to the latest information rather than only the training data.

### Limitations

There were several limitations to this study. The study included a limited number of topics (10 in total), which can only offer insight but cannot possibly cover the full spectrum of complexity and diversity within the two disciplines. Only ChatGPT 3.5 was prompted since it is the most widely used LLM for this purpose and has a free tier that the majority of users rely on. Newer models, including GPT-4, Claude+, and Google’s Gemini, may give significantly different results. Our study focused on the accuracy of citations and DOIs without an exploration of potential user feedback or subjective assessment of the overall quality and coherence of the generated content. These limitations can be addressed in future research.

### Conclusion

In conclusion, our study provides valuable insights into the performance of ChatGPT in generating citations and references across interdisciplinary domains. These findings contribute to the ongoing discourse on the use of LLMs in scholarly writing, emphasizing the need for nuanced consideration of discipline-specific challenges and the importance of robust validation processes to ensure the accuracy and reliability of generated content.

## Appendix

Multimedia Appendix 1 List of included topics and ChatGPT 3.5 prompt structure.

## Abbreviations

AI: artificial intelligence
BERT: bidirectional encoder representations from transformers
DOI: Digital Object Identifier
LLM: large language model
RNN: recurrent neural net

## Data Availability

The data sets generated during and/or analyzed during this study are available from the corresponding author upon reasonable request.
