# Supplementary material for: Evaluation of Large Language Model Performance and Reliability for Citations and References in Scholarly Writing: Cross-Disciplinary Study
Source: J Med Internet Res. 2024 Apr 5;26:e52935. doi: 10.2196/52935 (PMC11031695; doi:10.2196/52935)
Supplement: Multimedia Appendix 1 [file jmir_v26i1e52935_app1.docx]

Appendix 1

# **Topics Included**

| # | Topic | Category |
| --- | --- | --- |
| 1 | The feasibility of video game therapy in adolescent mood disorders: a philosophical exploration | Humanities |
| 2 | Human-Machine Collaboration: the future of AI Art beyond the hype | Humanities |
| 3 | Crime and Punishment, a modern interpretation of the Victorian England Criminal Justice System | Humanities |
| 4 | Aesthetic Experience and Human Evolution - Re-asserting Objective Standard of Artistic Excellence | Humanities |
| 5 | The Blurring Boundaries of the Human and Machine: Exploring the Aesthetic Implications in AGI Art. | Humanities |
| 6 | Undifferentiated Pancreatic Carcinomas, Clinical Features and Therapeutic Options: What We Know. | Natural Sciences |
| 7 | Risk Factors for Acute Rejection in Liver Transplantation and Its Impact on the Outcomes of Recipients | Natural Sciences |
| 8 | Bile is a Reliable and Valuable Source to Study cfDNA in Biliary Tract Cancers | Natural Sciences |
| 9 | Preoperative Nomogram for Microvascular Invasion Prediction Based on a Clinical Database in Hepatocellular Carcinoma | Natural Sciences |
| 10 | Extended Pancreato-Duodenectomy Coupled with Adjuvant Chemotherapy for SMARCB1/INI1 Deficient Pancreatic Carcinoma: A Case Report and Literature Review | Natural Sciences |

# **Sample Prompts**

Write an introduction section for a manuscript titled “[TOPIC HERE]”. Write in the context of a [CATEGORY HERE e.g philosophy] academic manuscript. Include references and citations of relevant published literature. Use the APA format for citations and references and include the DOI for each reference. Include at least 8 references.

*The model may be instructed to expound on the generated text with citations if necessary.*
